# Multimodal Autoencoder Predicts fNIRS Resting State From EEG Signals

**DOI:** 10.1007/s12021-021-09538-3

**Published:** 2021-08-10

**Authors:** Parikshat Sirpal, Rafat Damseh, Ke Peng, Dang Khoa Nguyen, Frédéric Lesage

**Affiliations:** 1 École Polytechnique de Montréal, Université de Montréal, C.P. 6079, Succ. Centre-Ville, Montréal, H3C 3A7 Canada; 2 Neurology Division, Centre Hospitalier de L’Université de Montréal (CHUM), 1000 Saint-Denis, Montréal, H2X 0C1 Canada; 3 Research Centre, Montréal Heart Institute, Montréal, Canada

**Keywords:** EEG-fNIRS, Functional brain imaging, Deep neural networks, Epilepsy, Resting state, Functional connectivity, Neurovascular coupling

## Abstract

In this work, we introduce a deep learning architecture for evaluation on multimodal electroencephalographic (EEG) and functional near-infrared spectroscopy (fNIRS) recordings from 40 epileptic patients. Long short-term memory units and convolutional neural networks are integrated within a multimodal sequence-to-sequence autoencoder. The trained neural network predicts fNIRS signals from EEG, sans a priori, by hierarchically extracting deep features from EEG full spectra and specific EEG frequency bands. Results show that higher frequency EEG ranges are predictive of fNIRS signals with the gamma band inputs dominating fNIRS prediction as compared to other frequency envelopes. Seed based functional connectivity validates similar patterns between experimental fNIRS and our model’s fNIRS reconstructions. This is the first study that shows it is possible to predict brain hemodynamics (fNIRS) from encoded neural data (EEG) in the resting human epileptic brain based on power spectrum amplitude modulation of frequency oscillations in the context of specific hypotheses about how EEG frequency bands decode fNIRS signals.

## Introduction

Functional near infrared spectroscopy (fNIRS) is a non-invasive, mobile, and cost-effective neuroimaging technology that uses near infrared light to continually monitor changes in cerebral hemodynamic parameters (i.e., oxygenated (HbO) and deoxygenated hemoglobin (HbR), and total hemoglobin (HbT)) (Jobsis, 1977). The fNIRS method relies on the neurovascular coupling phenomenon which describes the intimate spatial and temporal relationship between neural activity and cerebral blood flow to map acute functional changes in the brain (Girouard & Iadecola, 2006). In a typical fNIRS setup, optodes corresponding to near-infrared light sources and their complimentary detectors are placed on the surface of the subject’s head. Infrared light emitted from the light source is absorbed or scattered as it enters cerebral tissue. Detected light is used to calculate the blood oxygenation changes associated with cerebral hemodynamic activity using the modified Beer-Lambert law (Kocsis et al., 2006; Scholkmann et al., 2014). Concentration changes in the oxygenation of hemoglobin quantifies the absorption of infrared light by the brain.

The fNIRS method offers several advantages as an alternative or complement to other functional imaging techniques (i.e., fMRI) (Strangman et al., 2002). fNIRS offers increased temporal resolution as compared to fMRI, and fNIRS hardware can be integrated with other modalities such as scalp electroencephalography (EEG) (Fazli et al., 2012; Khan & Hong, 2017; Miller, 2012). fNIRS signals have been recently used in studying brain state decoding as well as proven useful for brain computer interfacing over the last decade (Hong et al., 2015; Khan & Hong, 2015).

Scalp EEG technology is the clinical gold standard for studying the human brain (Müller-Putz, 2020) and EEG recordings can be classified into specific frequency bands: alpha, beta, delta, gamma, and theta (Cho et al., 2014; Freeman et al., 2003; Pedregosa et al., 2011; Zhao et al., 2018). The delta frequency range encompasses low frequencies with relatively high amplitude and slow waveforms ranging from 0.25–3.0 Hz. Delta frequencies are common in normal sleep and may incidentally appear with focal lesions, metabolic encephalopathy, or hydrocephalus (Amzica & Steriade, 1998; Hofle et al., 1997; Knyazev, 2012). The theta band includes frequencies between 4 and 7 Hz. While normal in young individuals, the theta frequency envelope is interpreted as slow activity in awake adults (Mantini et al., 2007; Pizzo et al., 2016; Sitnikova et al., 2016). As with delta waves, theta waves may be seen in focal lesions or in a more generalized distribution in diffuse neurological disorders. Alpha frequencies are between 8 and 13 Hz, representing the dominant rhythm in awake adults (Koch et al., 2008; Sigala et al., 2014). Beta activity ranges in frequency between 14–30 Hz and is usually observed in a bilaterally frontal symmetrical distribution (Canolty et al., 2006; Freeman et al., 2003; Merker, 2016). Higher frequency ranges represent gamma wave oscillations between 30–100 Hz. Gamma activity is seen during a wide range of activities, and is enhanced in rapid eye movement during sleep (Gross & Gotman, 1999; Hughes, 2008).

Multimodal EEG-fNIRS experimental setups record the spatiotemporal dynamics of brain activity, provide opportunities to observe the population dynamics of neural ensembles and offer increased benefit in fundamental and clinical analyses (Goldman et al., 2002; Laufs et al., 2003; Martinez-Montes et al., 2004; McKenna et al., 1994; Salek-Haddadi et al., 2003). In such setups, scalp EEG measures the brain’s electrical activity, and fNIRS signals encode the brain’s hemodynamic response (Chiarelli et al., 2017; Ogawa et al., 1992), with a delay of approximately 3 seconds post neural activity. Data from EEG-fNIRS setups have established causality between neuronal firing and changes in HbO, HbR, and HbT, reflecting electrical and hemodynamic fluctuations dictated by neurovascular coupling (Hughes, 2008; Logothetis et al., 2001; Mukamel et al., 2005; Singh, 2012). Recent interest has focused on determining spatial hemodynamic correlates from EEG recorded activity, particularly, in the blood oxygen level dependent signal (BOLD) (Czisch et al., 2004; Lemieux et al., 2001; Lövblad et al., 1999). Resting state studies have successfully demonstrated that low frequency EEG band signals are negatively correlated with modulations in the BOLD signal, particularly, infra-low gamma EEG band envelopes (Jia & Kohn, 2011; Niessing et al., 2005; Sumiyoshi et al., 2012).

The characterization of the relationship between electrophysiology and cerebral hemodynamics is clinically relevant in epilepsy. Seizures are self-terminating paroxysmal representations of aberrant brain activity (Moshé et al., 2015). It is believed that the neurovascular machinery causing seizures is similarly present in the brain interictally during normal function, suggesting to some extent that epilepsy is a dynamic disorder (Kobayashi et al., 2006; Richardson, 2012). The resting epileptic brain displays spontaneous neural activity believed to reflect its functional organization (Rojas et al., 2018; Tracy & Doucet, 2015). The interdependence of each component (i.e., neural and vascular) is a topic of interest to the wider clinical and neuroscience community. fMRI studies have shown that resting state networks in the epileptic brain undergo changes in their functional architecture (Luo et al., 2011; Wang et al., 2011). Increasingly, “task-free” resting state conditions in fMRI studies have been conducted with the assumption that functionally connected brain networks show similar profiles of activity over time (De Luca et al., 2006; He & Liu, 2008; Niu & He, 2014; Palva et al., 2010; Richardson, 2012; Shen, 2015).

In the context of epilepsy, resting state fMRI studies have shown that functional networks are abnormal (Bettus et al., 2009; Honda et al., 2021; Tracy & Doucet, 2015; Zhang et al., 2009, 2010a, b). Pre-clinical studies have proposed that there is a correlation between slow fluctuations in the resting state BOLD signal (~0.1 Hz) and slow fluctuations in neuronal firing rates in gamma band local field potentials (Richardson, 2012; Shmuel & Leopold, 2008; Zhang et al., 2020). This suggests that the resting state is related to physiologically active dynamic neuronal processes. Utilizing fNIRS signals for resting state functional connectivity has gained attention as a promising imaging tool to study brain function and provide valuable insight into the intrinsic networks present within the human epileptic brain (Fishburn et al., 2014; Geng et al., 2017; Niu & He, 2014; Wang et al., 2017).

In this study, we hypothesize that we can predict brain hemodynamics from electrical signals using a deep learning architecture from resting state multimodal EEG-fNIRS recordings collected from a cohort of 40 epileptic patients. Following which, we hypothesize that functional connectivity patterns derived from higher EEG frequency envelopes are increased as compared to lower EEG frequency envelopes.

## Methods

### Subjects and Protocol

Forty patients (27 males, 13 females; ranging in age of 11 to 62 years in age; mean age of 32.42 years, and standard deviation of 13.97 years) with refractory focal epilepsy were recruited for prolonged EEG-fNIRS recordings. Epilepsy diagnosis and epileptic focus localization was based on a comprehensive evaluation which included clinical history, video-EEG recording of interictal spikes and seizures, magnetic resonance imaging (MRI), positron emission tomography (PET) and for some patients ictal single photon emission computed tomography (SPECT) and magnetoencephalography (MEG) scans. Full details regarding patient profiles including age, gender, EEG and MRI findings are found in Table 1 of (Peng et al., 2014; Sirpal et al., 2019). A subset of patients had MRI evidence of encephalomalacia, cortical dysplasia, and/or hippocampal atrophy, a common finding in epilepsy, but this was neither an inclusion nor exclusion criterion. The presence of such findings (Dhamija et al., 2011; Woermann & Vollmar, 2009) is a common MRI finding in epileptic brains. The institutional review boards of Sainte-Justine Hospital and Centre Hospitalier de l’Université de Montréal approved the study.

**Table 1:** Detailed overview of the proposed convolutional neural network long-short term autoencoder (CNN-LSTM AE) model. The network receives as input resting state EEG time-series sequences, represented as a single matrix, and is trained to reconstruct the corresponding fNIRS resting state output. Model specifications and hyperparameters were heuristically determined. Convolutions and deconvolutions have kernels of size (1,2), and thus their effect is along the time dimension. Convolutions help in generating embeddings with higher level abstraction of the input EEG sequence. Deconvolutions reconstruct the fNIRS sequence at full resolution based on output embeddings. The decoder and encoder LSTM units have ReLU (Rectified linear units) activations

Layer	Description	Output size
Input	EEG sample sequence	(EEG sequence length, number of time points is 500, number of EEG channels is 21)
EEG Sequence Embedding		
2-Dimensional convolution + Average Pooling + Dropout	$2D convolution: stride=(\mathrm{1,2});$ $kernel size=(\mathrm{1,7});$ $ReLU activation.$ $Dropout: 20\%.$ $Average Pooling kernel: (\mathrm{1,2})$	(EEG sequence length, 125, number of Features Maps)
2-Dimensional convolution + Average Pooling + Dropout	$2D convolution: stride=(\mathrm{1,2});$ $kernel size=(\mathrm{1,7});$ $ReLU activation.$ $Dropout: 20\%.$ $Average Pooling kernel: (\mathrm{1,2})$	(EEG sequence length, 62, number of Features Maps)
Reshape	Reshape into an elongated tensor	(EEG sequence length, 62 * number of Features Maps)
Encoder		
LSTM 1 + Dropout	An LSTM layer with number of cells equal to the number of EEG sequence length. ReLU activation. Dropout: 20%	(EEG sequence length, 512)
LSTM 2 + Dropout	An LSTM layer with number of cells equal to the number of EEG sequence length. ReLU activation. Dropout: 20%	(1, 256)
Decoder		
Repeat	Create repeated version of the latent vector	(fNIRS sequence length, 256)
LSTM 3 + Dropout	An LSTM layer with number of cells equal to the number of EEG sequence length. ReLU activation. Dropout: 20%	(fNIRS sequence length, 312)
LSTM 4 + Dropout	An LSTM layer with number of cells equal to the number of EEG sequence length. ReLU activation. Dropout: 20%	(fNIRS sequence length, 695)
fNIRS Sequence Reconstruction		
Reshape	Reshape into a 2D tensor	(fNIRS sequence length, 5, number of Feature Maps 3)
Deconvolution1 + Dropout	$2D deconvolution: stride= (\mathrm{1,2}); ReLU activation;$ $kernel size = (\mathrm{1,2}). Dropout: 20\%.$	(fNIRS sequence length, 10, number of Feature Maps 4)
Deconvolution2 + Dropout	$2D deconvolution: stride=(\mathrm{1,2}); ReLU activation;$ $kernel size=(\mathrm{1,2}). Dropout: 20\%.$	(fNIRS sequence length, 20, number of fNIRS channels)

#### EEG-fNIRS Data Acquisition and Pre-Processing

**Fig. 1:**
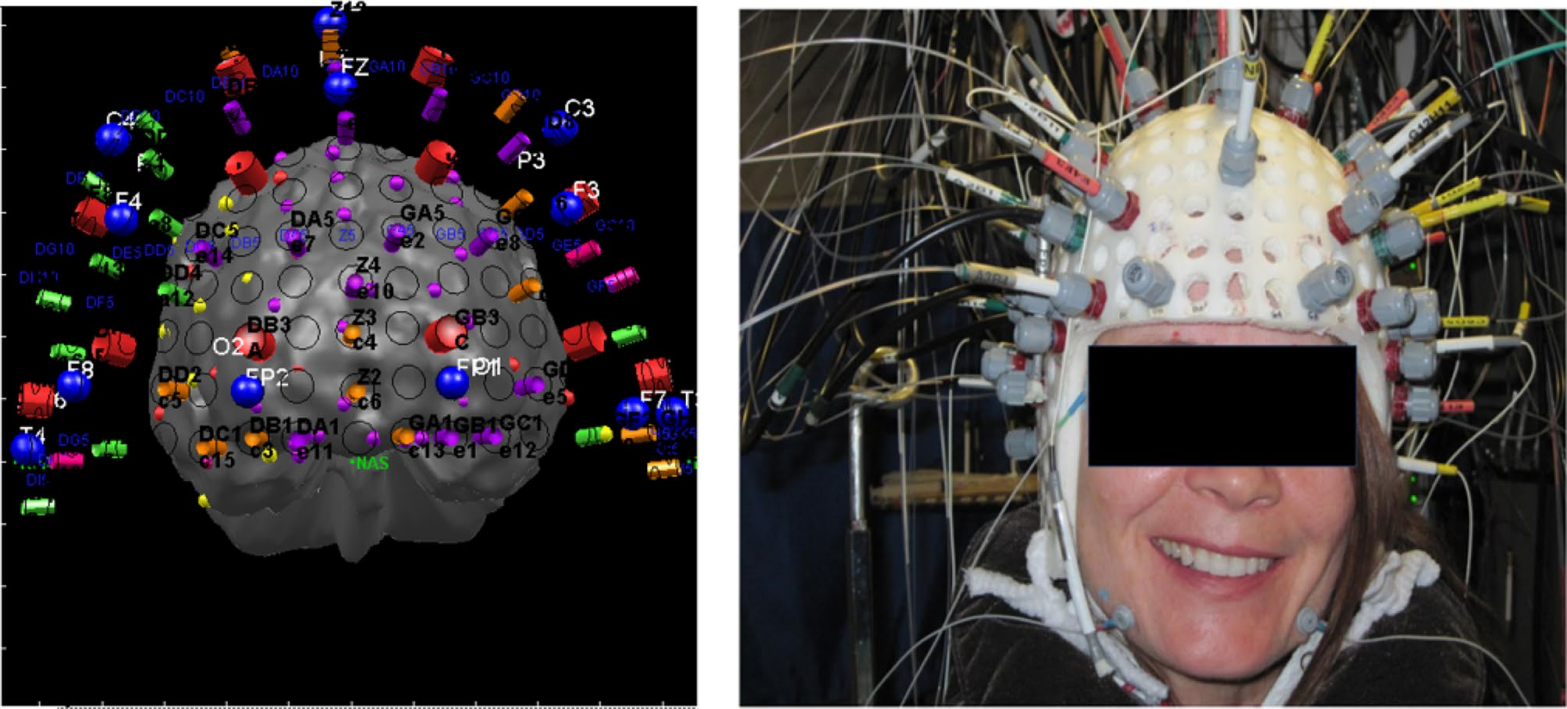
EEG and fNIRS channel-configuration and custom-made multimodal EEG-fNIRS helmets used for EEG and fNIRS data acquisition. Helmets of different sizes and shapes to fit patients’ head comfortably were made from plastic and polyvinyl chloride making them rigid and light. The EEG-fNIRS configuration allows for full head coverage and follows the 10–20 EEG placement system. The fNIRS channel configuration, as well as the EEG (which are in blue dots), are superimposed on the patient’s MRI. We used a 3D camera and stereotaxic system (Frameless 39 from Rogue research) to determine the 3-D coordinates of the optodes relative to the patient’s anatomical MRI

Continuous EEG-fNIRS recordings were performed at the Optical Imaging Laboratory of Sainte-Justine Hospital in Montréal, Canada. Experimental protocol ensured ambient noise and lights were maintained at a minimum for patient comfort during data acquisition. Patients were further instructed to remain calm and placed in comfortable, climate-controlled ambulatory suites with curtains drawn to limit ambient light. Patients were continually telemonitored by trained clinical staff. fNIRS data was collected using the Imagent Tissue Oximeter system (ISS Inc.), a multi-channel frequency domain system recording at 19.5 Hz with wavelengths of 690 nm and 830 nm for sensitivity to HbR and HbO respectively. EEG data was recorded according to the standard 10–20 system using 21 electrodes (in positions *Fp1, Fp2, F7, F3, Fz, F4, F8, T7, C3, Cz, C4, T8, P7, P3, Pz, P4, P8, O1, O2*) at 500 Hz (Neuroscan Synamps 2TM system). Custom-made helmets, taking into consideration different head sizes and shapes were made to fit comfortably. Plastic and polyvinyl chloride manufacturing materials made them rigid and light. The helmets were equipped with a total of 64 light sources, 16 light detectors and 19 EEG electrodes that allowed for stable optical coupling between cortical regions and the scalp. This further prevented inter-optode shifting and movement artifacts to a large extent. Sensitivity of near-infrared light to cortical tissue was maintained by positioning the optodes approximately 3–4 cm apart. Electrodes were placed following the 10–20 EEG instrumentation standard, allowing for full head coverage (Barlow et al., 1974). Figure 1 shows the EEG-fNIRS helmet placed on a patient’s head.

EEG data was bandpass filtered between 0.1–100 Hz to remove instrumental noise and to remove drift related to physiological activity, particularly, higher frequencies. The unprocessed raw time series of the HbO and HbR signals was bandpass filtered to remove specific frequency components attributed to cardiac (approximately 1 Hz) and/or respiratory activity (approximately 0.2–0.3 Hz) (Gramfort et al., 2014; Lu et al., 2010; Peng et al., 2014). Signal fidelity was examined prior to analysis by channel-wise verification of signal intensity. Bandpass filtering was applied to EEG data to compute frequency bands of interest. We used a FIR bandpass filter and the lowcut and highcut values (Hz) for the delta, theta, alpha, beta, and gamma frequencies were set as: [1, 4], [4, 8], [8, 12], [12, 30], [30, 100] respectively (Gramfort et al., 2014). The signal to noise ratio (SNR) threshold applied in channel analysis was defined as those channels less than 30% of the mean SNR of all channels. fNIRS channels deemed to have SNR were eliminated and not included for analysis. This led to an average of 138 channels per patient. Changes in HbO and HbR were calculated via the HomER and MNE software packages (Gramfort et al., 2014; Huppert et al., 2009).

Multiple consecutive recordings were performed, with each recording approximately 15 min led to a compendium of 200 recordings totaling 50 hours of recording time. Data was bandpass filtered in the 0.01 to 0.1 Hz frequency range to be in the resting state range (Tong et al., 2012). The resting state period (indexed from patients when they were resting comfortably) ranged between 7 to 10 minutes with a mean of 8.35 minutes (Geng et al., 2017; Li et al., 2015; Zhang et al., 2010a, b). To correct for motion, we performed dimension reduction via principal component analysis on EEG-fNIRS data and removed components with the most variance. Further, artifact rejection with (10% variation from normalized intensity) was applied to remove additional motion artifacts. Artifact-free data points were then filtered for the effects of respiratory and cardiac signal with a cutoff frequency of 0.2 Hz. Finally, HbO concentrations were calculated for each channel using the modified Beer–Lambert law.

Structural MRI registration of optode and electrode position was done using neuro-navigation (Brainsight, Rogue-Research Inc.). Channel positions were cross-referenced with the patient MRI and adapted to ensure coverage of the epileptic focus, the contralateral homologous region, and as much area as possible of other brain regions. The MRI was segmented into six different layers: air, scalp, skull, CSF, gray matter and white matter. The gray matter layer was used to extract six two-dimensional cortical projections. The three-dimensional position of each channel was projected onto these two-dimensional topographic maps, of which the following views were considered: dorsal, frontal, left and right views.

### Neural Network Architecture

We built a deep sequence-to-sequence multimodal autoencoder to predict fNIRS signals from input scalp EEG signals. Autoencoders are powerful machine learning models trained in a self-supervised fashion to reconstruct inputs by learning their abstract representations (Kocsis et al., 2006; Lindauer et al., 2010; Socher et al., 2011; Vincent et al., 2010). The autoencoder embedded signals in a low dimensional latent space, where both the encoder and decoder are formulated as deep neural networks.

Recurrent neural networks (RNN) have been widely used in time series modeling since they account for the temporal state within data (Baytas et al., 2017; Chung et al., 2016; Merity et al., 2018; Mikolov et al., 2010). The output depends on hidden states and feedback connections present within hidden units. Previous states can be used as inputs, thereby allowing RNNs to hold memory. In our model, we used backpropagation through time, a common gradient descent type training technique (Sutskever et al., 2014). The innate problem of RNN gradient based training is that derivatives propagated via recurrent connections either become exceedingly small or large (Goodfellow et al., 2016; Luong et al., 2015), causing a vanishing or exploding gradient respectively. Long short-term memory units (LSTM), a variant of the vanilla RNN architecture overcomes the vanishing gradient problem (Greff et al., 2017; Gregor et al., 2015; Lecun et al., 2015). LSTM units receive external inputs and generate hidden outputs via input, output, and forget gates and a memory cell. The gates and memory cell are internally connected with weighted links. The gates are connected with external sources, which are current state sequential inputs and previous hidden states. This prevents the LSTM from storing useless or noisy input information (Greff et al., 2017; Gregor et al., 2015; Lecun et al., 2015).

The LSTM autoencoder model (LSTM-AE) as proposed by Srivastava et al. consists of encoder LSTM units and decoder LSTM units (Srivastava et al., 2015). The encoder LSTM receives input sequences and encodes them into a feature vector as the LSTM generates hidden outputs (Lipton et al., 2016; Wang et al., 2016). Likewise, the decoder LSTM receives the feature vector and decodes it into the original input sequences. LSTM-AEs learn a compressed representation of sequential data and have been used in video, text, audio, and time series sequence data (Lipton et al., 2016; Srivastava et al., 2015; Wang et al., 2016). In this work, multiple LSTM layers were incorporated to learn temporal representations. Our model also includes convolutional layers to extract high level spatial percepts from channel combinations. We input EEG sequential data accounting for hemodynamic delays to perform sequence-to-sequence encoding (Luong et al., 2015; Truong et al., 2018; Vincent et al., 2008; Zhang, 2018). These input EEG sequences are convolved by two convolutional neural networks (CNN) and subsequently fed into the first two encoding long short-term memory (LSTM) modules. EEG data samples are projected in the latent space with fixed length vectors that provide more compressed representations, which are then used to decode and reconstruct the output fNIRS data, by the LSTM decoding modules.

After testing multiple architectures with exhaustive hyper-parameter optimization, we designed our model as follows: The encoder is comprised of LSTM layers preceded by convolutional blocks. Convolutions in each block have a kernel size of (1, 7) and stride size of (1, 2). The decoder is comprised of LSTM layers which manipulate the vectors in the latent space to provide a final output dimension equal to that of an fNIRS sample. We evaluated our model in terms of cross-modal reconstruction error (Zhao et al., 2018), denoted as RE. The objective is to simultaneously minimize the distance between fNIRS data samples and maximize the distance between each fNIRS and EEG data points (i.e., minimizing the RE is equivalent to maximizing the likelihood function). Once the model was trained, the corresponding RE was calculated on an independent testing subset (see below) by computing the sum of the Euclidean distance between ${x}_{t}$ and its corresponding reconstruction, $\widehat{x}$, over all L dimensions, as expressed in Eq. 1 below:

1 $${\in }_{t}=\sum_{l=1}^{L}\left|{\widehat{x}}_{t,l}-{x}_{t,l}\right|, t\epsilon\; T$$

Model output is denoted as ${\widehat{|x}}_{t}|$.

**Fig. 2:**
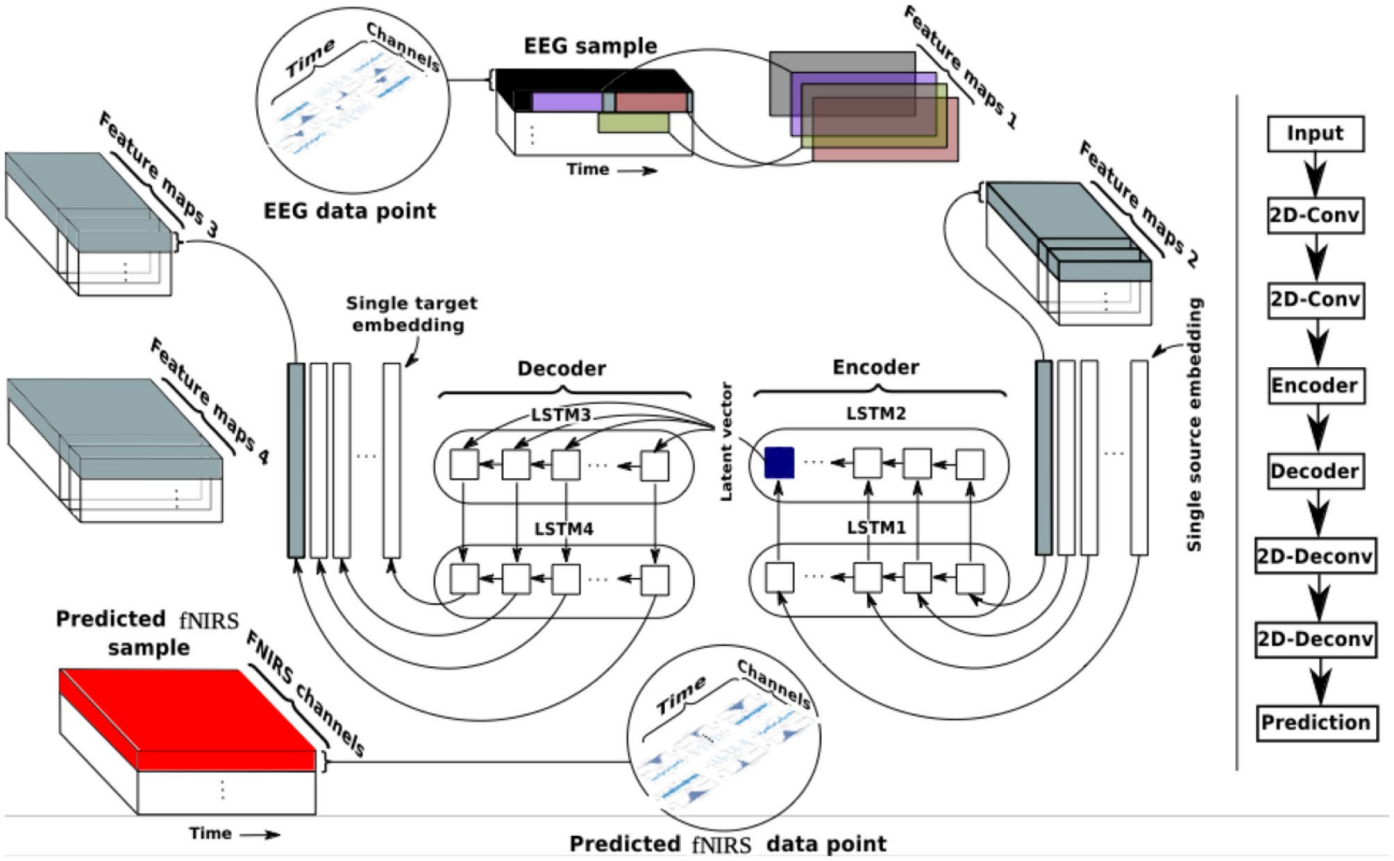
Multimodal EEG to fNIRS reconstruction using our patient specific sequence to sequence LSTM autoencoder model. Given EEG input data into the encoder, the model decodes and reconstructs fNIRS output. After data collection and resting state segment annotation, data processing and model development, the data is finalized as a 4-D tensor with shape: (samples per batch, sequence length, time points, and channels). The model has encoder and decoder compartments, each with 2 LSTM layers, determined heuristically. Table 1 below provides details of the model

EEG data is processed as follows. First, matching EEG and fNIRS data are parsed from our data directory, following which the respective data (EEG or fNIRS) is labeled according to the resting state periods. Feature scaling is performed using the MinMaxScaler class (Pedregosa et al., 2011) on EEG input data which sets the range of values between 0 and 1. Input signals are mean centered prior to being fed into the model. Then, data is fed into the convolutional layers and travels to the LSTM and deconvolution modules. A detailed schematic view of our model is shown in Fig. 2 below.

### Training Details

The model was designed to use patient specific EEG signals as input to decode fNIRS signals. For each patient, the data was randomly split into training, validation, and testing subsets, with a proportion of 60% training, 20% testing and 20% respectively. We experimented with various model depths and determined deep LSTMs to outperform shallow LSTMs. This is likely due to the larger hidden state which occurs because of increasing layers. Complete training details are given below.

- We initialized the LSTM’s parameters with the uniform distribution between 0 and 1. This was done to counteract the exploding gradients problem intrinsic to LSTMs, thereby enforcing a hard constraint on the norm of the gradient by scaling it between 0 and 1. Simultaneously, we specified starting node values for the LSTM computations by preparing a feed dictionary which has input EEG data and a target label. It is important to note that the LSTM can learn how to map input sequences as model training is patient specific into a fixed dimensional vector representation and can learn temporal dependencies.
- Backpropagation through time was used with a learning rate of 0.05, batch size of 60 and 50 epochs, all of which were heuristically determined.
- Each fNIRS signal generated corresponds to an EEG sequence input. An element in the EEG sequence corresponds to 1 second of recording with 500 time points (sampling frequency is 500 Hz) for each EEG channel. Data batches were generated for sequence processing by using the utility class for batch generation in the Keras framework. Briefly, this class uses as input a sequence of data points to produce batches for training and validation. Data points outside of the start and end indices of resting state periods (as marked in our ground truth) are not used in the output sequences. The final EEG data used as input is two dimensional, i.e., [***data points, channels***]*.*

To summarize, the model was trained as follows: **(a)** we designed LSTM layers with corresponding LSTM cells **(b)** model parameters were uniformly initialized in the range between [0,1], **(c)** dropout was applied with value of 0.2, and average pooling was applied to reduce the probability of model overfitting, **(d)** we used backpropagation through time with a learning rate of 0.05, **(e)** we used a batch size of 60 and 50 training epochs for each patient.

#### Model Validation

**Fig. 3:**
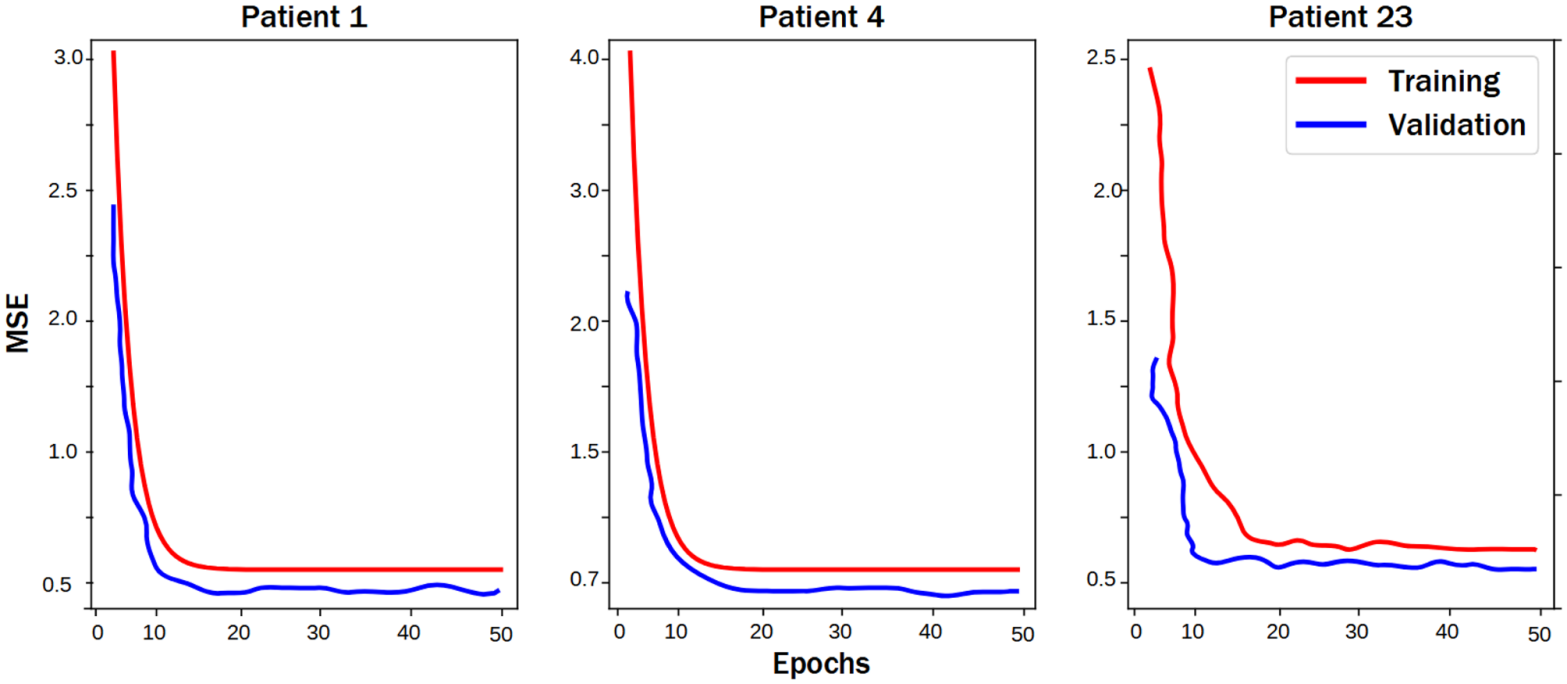
Learning curves are generated for the training and validation sets. The training and validation loss decrease to a point of stability with a minimal gap between the two final loss values. We note that the validation loss decreases to a point of stability with a minimal gap between the two final loss values. We note that the validation loss decreases to a point of stability and has a small gap with the training loss, mean squared error (MSE)

After training and saving our model’s weights, we validated the model’s intra-patient predictive capacity by using individual EEG recordings as input to predict fNIRS signals. This was possible since our dataset contains multiple recordings from each patient. To diagnose performance, we plotted learning curves to ensure we did not overfit during training. As an illustrative example, Fig. 3 shows the learning curves for patients 1, 4, and 23.

#### Model Predictions

The model predicts signals by appending ‘output state’, and ‘output prediction’ matrices. LSTM cells are connected recurrently to each other. Decoder inputs are two-dimensional matrices which are passed into decoder LSTM layers. fNIRS data is shifted one sequence ahead to hold data in LSTM memory and finally decoder outputs are returned due to the data passing through the deconvolution layers.

### Functional Connectivity Validation

We chose the seed channel from a region of interest, defined to be a region which had adequate optode coverage confirmed by our source/detector montage and an acceptable level of signal fidelity. That is, signals that were $\pm$ 2 standard deviations of the mean and displayed low SNR (i.e., signal amplitude less than 30% of mean signal amplitude) were removed from analysis. We then computed the Pearson product-moment correlation coefficients between the experimental fNIRS timeseries of the seed channel and the experimental fNIRS timeseries of all other channels. Subsequently, the Pearson product-moment correlations (and corresponding Fisher z-scores) were computed between the experimental seed channel timeseries and our model’s predicted fNIRS timeseries for all other channels. The two sets of correlation coefficients were respectively projected to an MRI head template based on the three dimensional coordinates of the corresponding channels using Atlasviewer (Aasted et al., 2015). The connectivity value at each voxel of the cortex was obtained from the correlation coefficients of all channels with a weighted-average method using the reciprocal of the cube of the distance from the voxel to each fNIRS channel.

In order to quantitively evaluate and compare the results of our functional connectivity studies, we computed the root mean square error (Eq. 2) i.e., the standard deviation of the residuals between functional connectivity values in experimental fNIRS and reconstructed fNIRS time courses derived from full spectrum EEG and specific EEG frequency band signals for all patients in our cohort.

2 $${RMSE}_{{FC}_{C}}=\sqrt{\frac{{{\sum }_{i=c}\left({fc}_{i}-{\widehat{f}}_{c}\right)}^{2}}{C}}$$

where *“C”* is the number of channels per functional connectivity analysis, ${fc}_{i}$ is the connectivity value of experimental fNIRS and ${\widehat{f}}_{C}$ is the connectivity value of model fNIRS reconstructions.

## Results

This section describes the reconstruction results obtained using full spectrum EEG and subsequently EEG frequency ranges as model input. Intra-patient reconstructions are also presented; we explore spatial reconstruction, resting state predictions, and functional connectivity.

### Full Spectrum EEG Performance and Feature Analysis

**Fig. 4:**
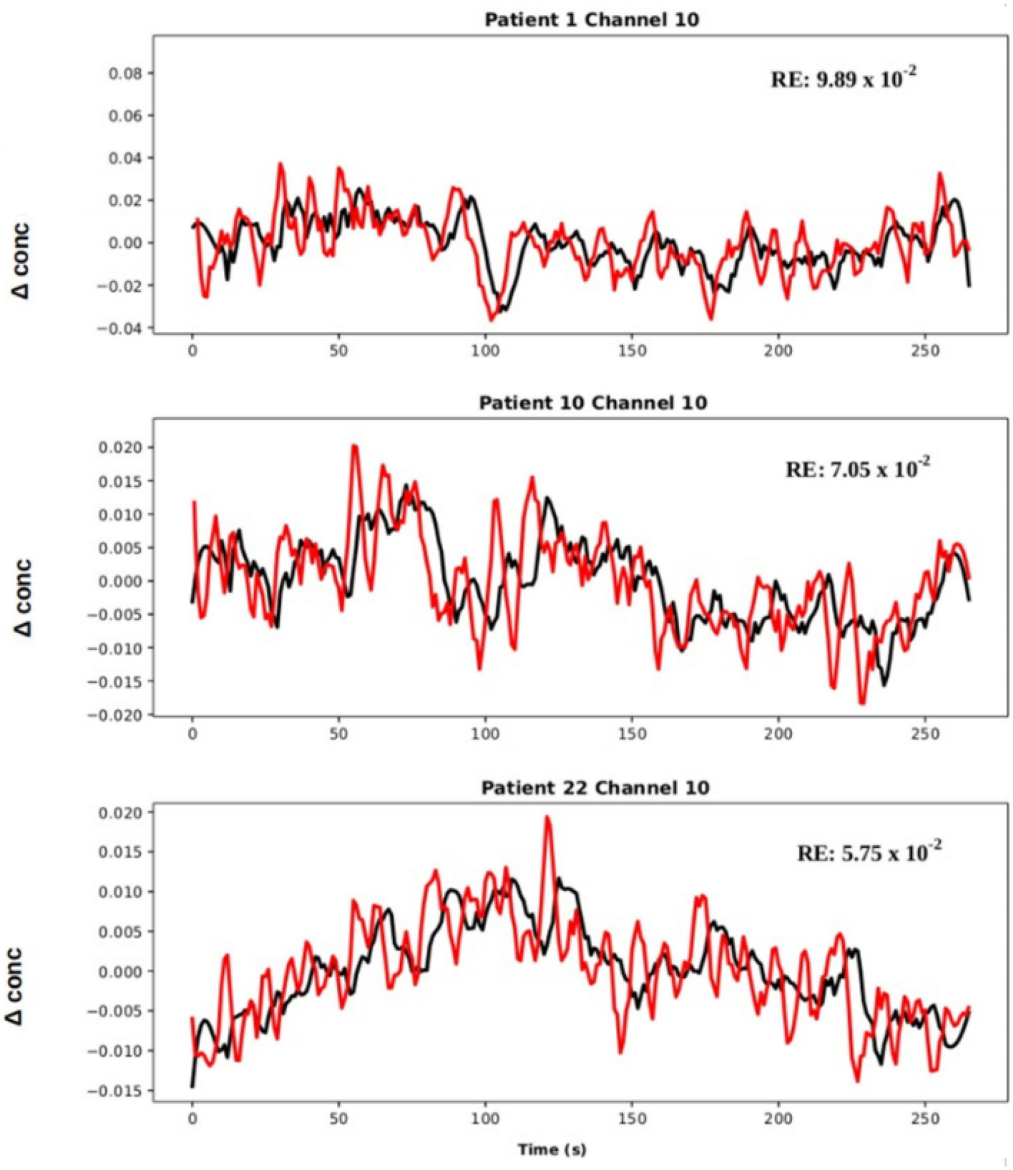
Decoded predictions of hemodynamic signals from cerebral electrical activity. Full spectrum EEG signals from all channels were used as input. fNIRS HbO reconstructions are shown from 3 patients in channel 10 (Channel 10’s SNR was adequate, located on the left temporal lobe). Black and red curves correspond to experimental and reconstructed fNIRS signals respectively. Data from patient 22 reconstructed with the lowest reconstruction error, RE, while patient 10 had the highest. The data has been mean centered and baseline is near zero, 250 s is shown here to illustrate seizure free, resting state periods. Note that the model accounts for the delay between EEG and fNIRS and the model fNIRS predictions are indicative of this delay

Resting state full spectrum EEG signals from all channels were input in the model. To decode fNIRS channels from encoded EEG channels, the model’s decoder layers used the encoder’s latent state as input as data traveled through LSTM units. Figure 4 below quantifies performance on selected individual patients with full spectrum EEG signals as input.

**Fig. 5:**
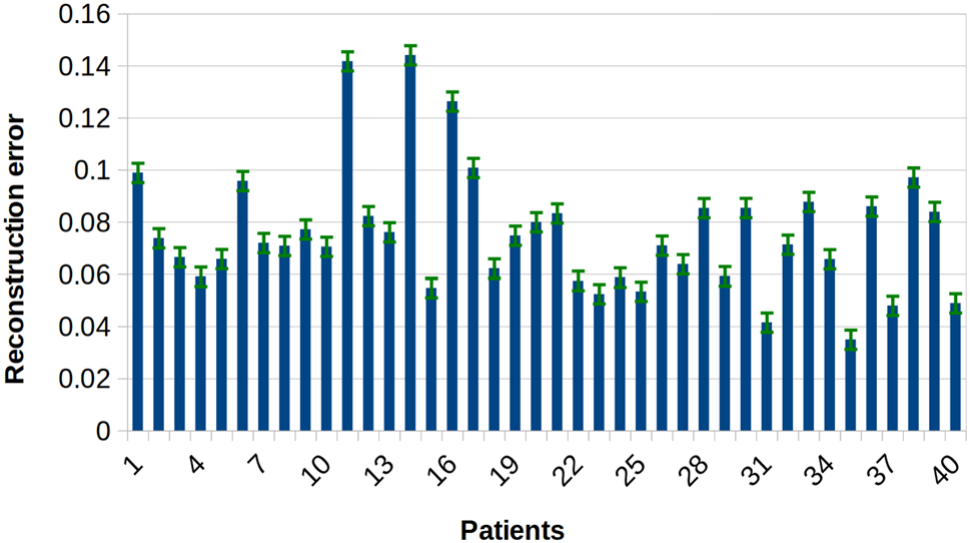
Full spectrum EEG to fNIRS reconstructions. The group estimate of full spectrum EEG signals from all channels were used as input in our network architecture, to reconstruct full fNIRS signals from all fNIRS channels

Figure 5 provides the group estimate of reconstruction error for all patients given scalp full spectrum EEG recordings.

#### Intra-patient Reconstructions on Separate Recording Sessions

**Fig. 6:**
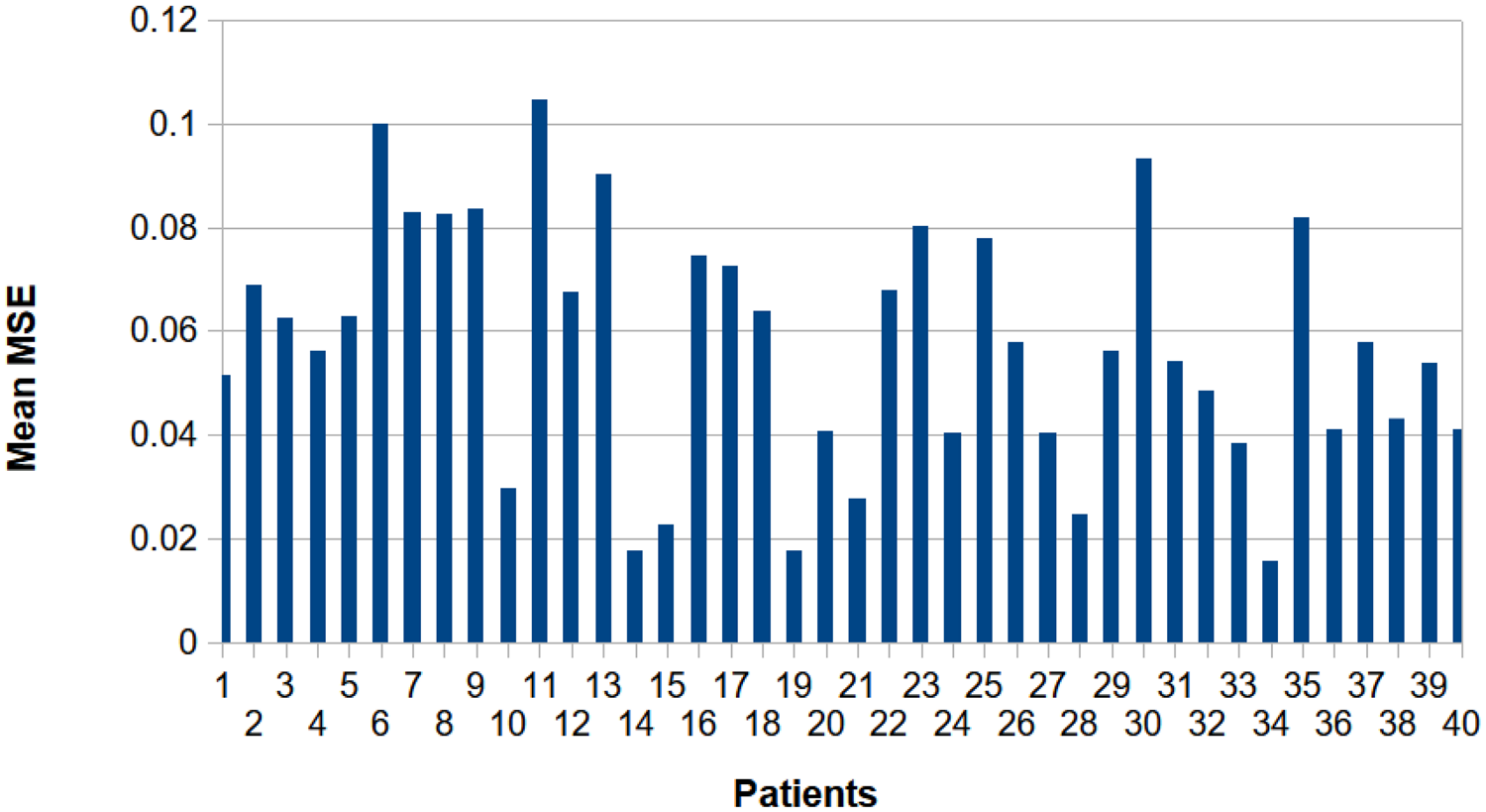
Group results for intra-patient fNIRS reconstructions. The network was trained on a patient’s single recording. Next, the network reconstructed subsequent recordings from the same patient. The data was partitioned into training, testing, and validation subsets in a 60/20/20 manner. This was done for all data across all patients and recordings

**Fig. 7:**
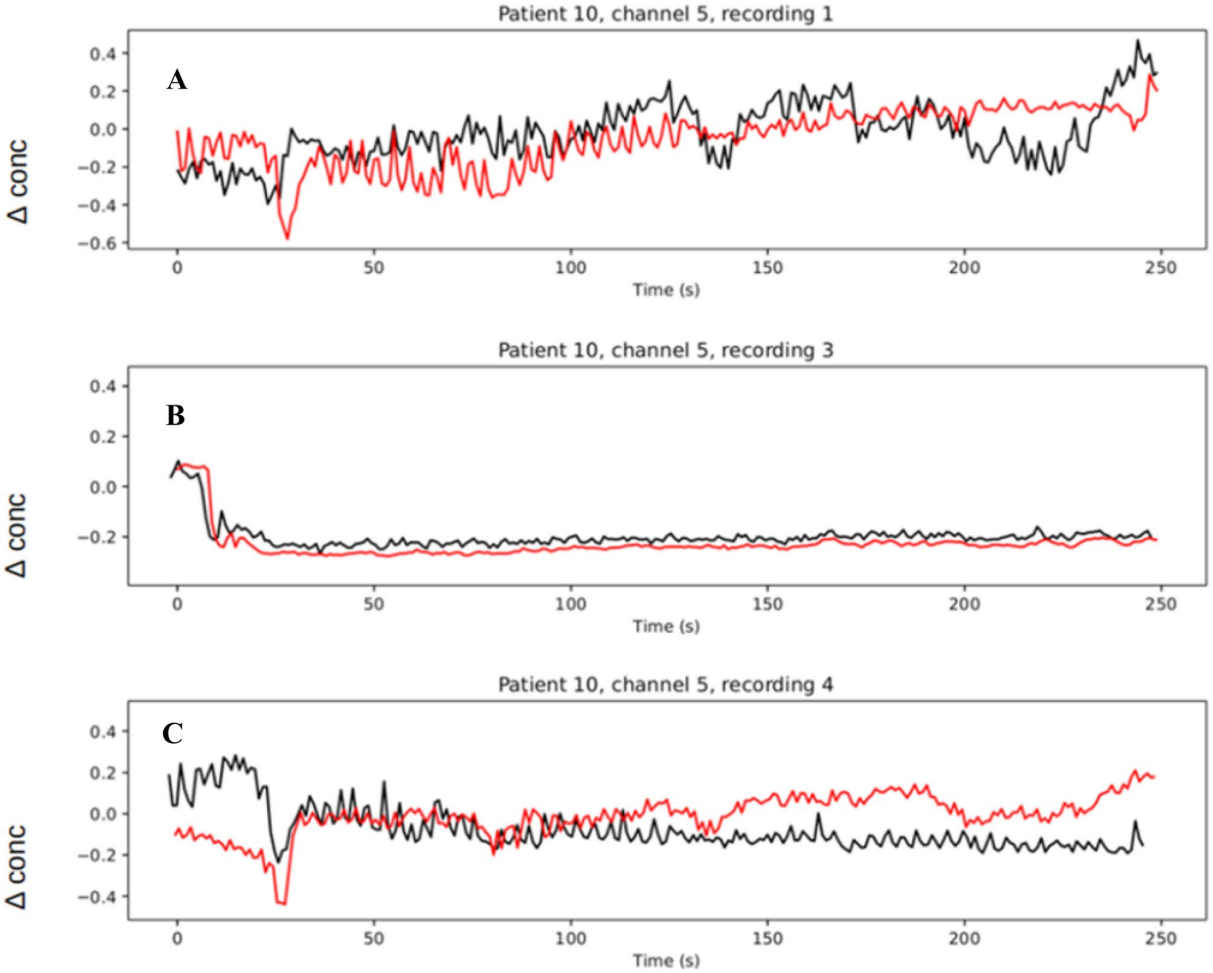
Intra-patient predictions of hemodynamic signals from cerebral electrical activity. A full spectrum resting state EEG single recording (patient 10 all channels) was used to train the model. After training, we saved the model weights and used as input a subsequent recording from the same patient. fNIRS reconstructions are shown here from 3 such recordings. Panels “**A**”, “**B**”, and “**C**” show the respective reconstruction of channel 5 from recordings 1, 3, and 4 (patient 10). The reconstruction error is 2.98 × 10^–2.^ Black and red curves correspond to experimental and reconstructed fNIRS signals respectively

Here, we report results on intra-patient fNIRS reconstructions provided EEG resting state as input. Specifically, we hypothesized that our model when trained with a patient’s single recording was able to reconstruct fNIRS signals from a subsequent recording. To examine our model’s predictive capacity and to cross-validate our model, we first trained our network on a patient’s single recording. Next, we used our trained network and aimed to reconstruct fNIRS signals from a subsequent recording from the same patient. The data was partitioned into training, testing, and validation subsets in a 60/20/20 manner. This was done for all data across all patients and recordings. Figure 6 displays the group results for intra-patient fNIRS signal reconstructions and Fig. 7 displays the fNIRS reconstructions for channel 5 from patient 10 across recordings 1, 3, 4.

#### Spatial Variability of Reconstructions

**Fig. 8:**
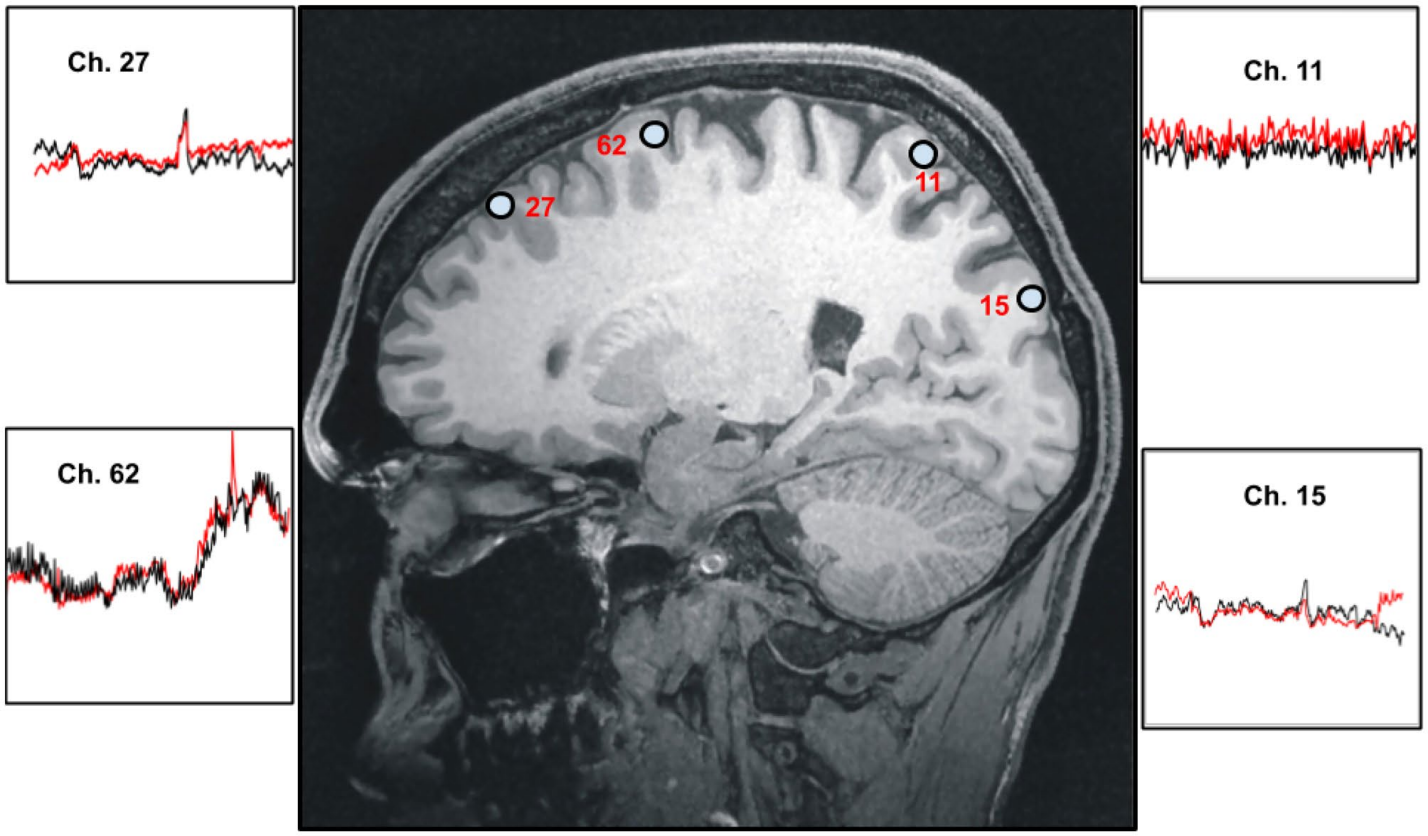
fNIRS spatial reconstructions, patient 10. To illustrate our network’s fNIRS reconstructions spatially, signals from multiple EEG channels are used as input, for which the locations are shown on the brain (blue circles). Reconstruction error ranges from $6.41x{10}^{-1}$ (channel 11) to $7.83x{10}^{-3}$ (channel 62), with the mean RE being $6.52x{10}^{-2}$ for all reconstructions. Black and red curves correspond to experimental and reconstructed fNIRS signals respectively

We then explored the model’s predictions sensitivity to channel location on the head. The topographic robustness of the model suggests the predictions are reasonably invariant across the brain. Channel locations were chosen if they offered coverage of most of the brain within the constraints of the source/detector montage and had an acceptable level of signal fidelity as indicated in “EEG-fNIRS Data Acquisition and Pre-Processing” section. As an illustrative example, Fig. 8 shows the model’s spatial predictions for patient 10.

### EEG Frequency Decomposition and Resting State Predictions

After model training and validation, we computed EEG frequency bands, namely: delta [0.5–3 Hz], theta [4–7 Hz], alpha [8–13 Hz], beta [14–30 Hz], and gamma [30–100 Hz]. To ensure the presence of appropriate power in the frequency ranges, the spectral power of EEG signals was obtained using the Welch’s power spectral density function. Welch's method was preferred over other methods (i.e., standard periodogram spectrum estimation and Bartlett's method) as Welch’s method offsets a reduced frequency resolution with a reduction in signal noise in the estimated power spectra in exchange for reducing the frequency resolution (Welch, 1967). The Welch method partitions the signal into overlapping segments thereby mitigating the loss of edge data. The overlapped data segments are then windowed in the time domain. Subsequent computation includes the discrete Fourier transform, followed by averaging the periodograms leading to a final $n x m$ array representing power measurements by frequency bins.

**Fig. 9:**
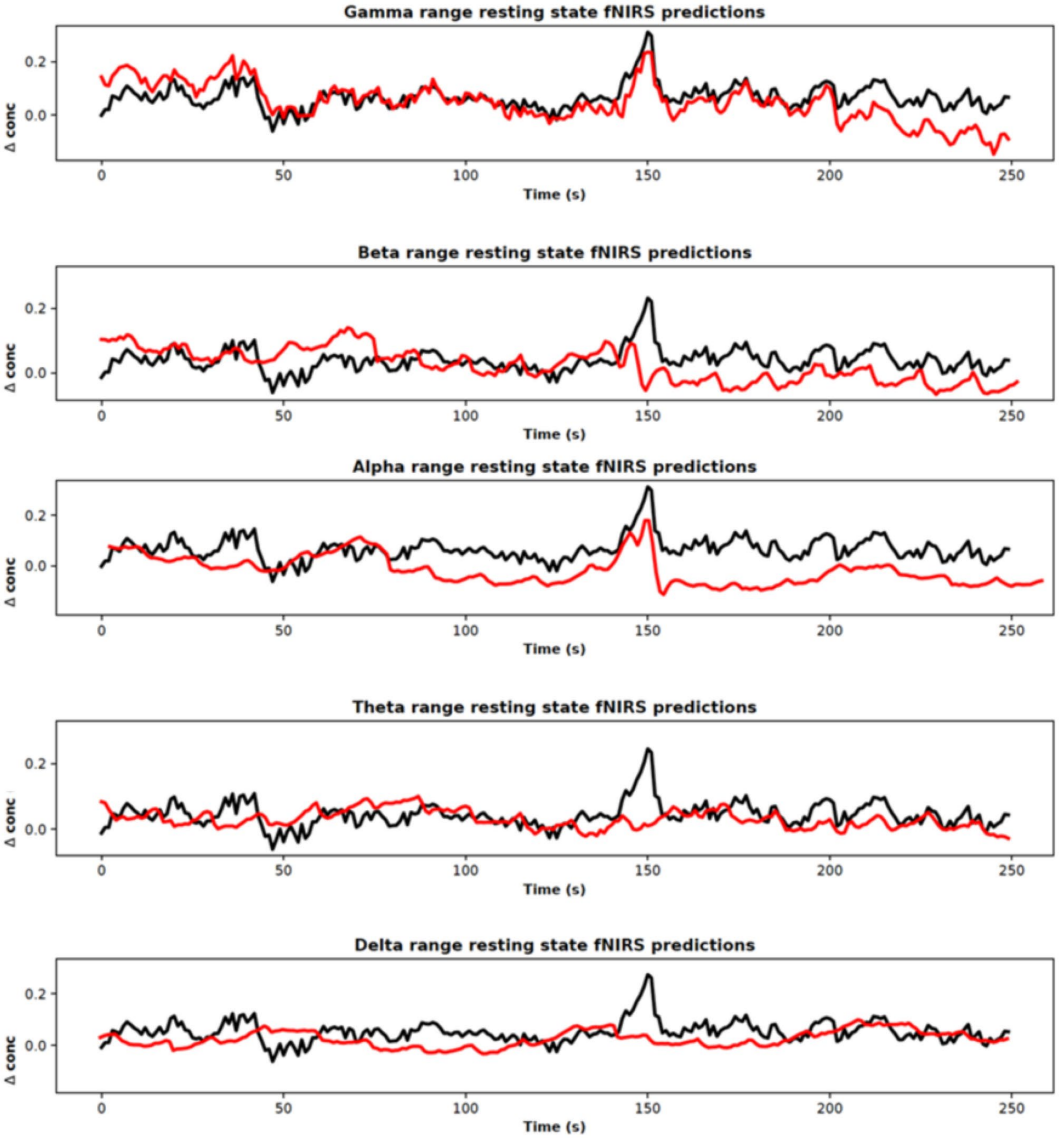
Resting state fNIRS predictions given EEG frequency range input, patient 10, channel 10. We obtained predicted fNIRS reconstructions given filtered EEG input for the following frequency bands: Delta: 0–3 Hz; Theta: 4–7 Hz; Alpha: 8–13 Hz; Beta: 14–30 Hz; Gamma: 30–100 Hz. Black and red curves correspond to experimental and reconstructed fNIRS signals respectively. We used a constant experimental fNIRS signal for comparison. The gamma range, which contains the greatest number of EEG frequencies reconstructs with more fidelity compared to ranges with less frequency components

All computations (including Fourier decomposition, Welch’s power spectral density) were performed using the MNE software package (Gramfort et al., 2014). Figure 9 shows the model’s predictions from EEG frequency ranges input using patient 10 (fNIRS channel 10).

**Fig. 10:**
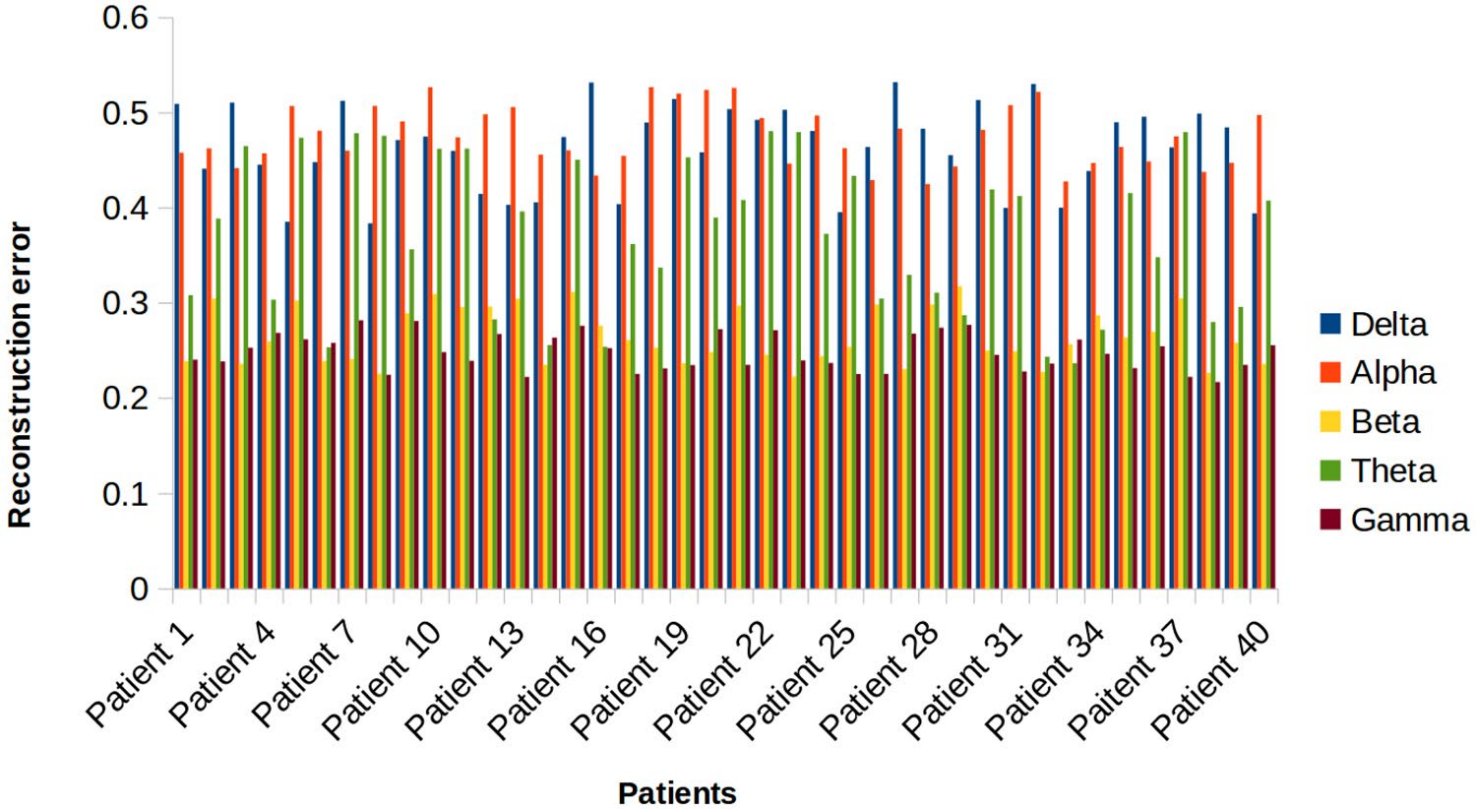
fNIRS reconstruction error given specific EEG frequency ranges for all patients, all channels. We obtained predicted reconstructions given filtered EEG input for the following frequency ranges: Delta: 0–3 Hz; Theta: 4–7 Hz; Alpha: 8–13 Hz; Beta: 14–30 Hz; Gamma: 30–100 Hz. The gamma range, which contains the greatest number of EEG frequencies, reconstructs with more fidelity and lowest reconstruction error metrics compared to ranges with less frequency components

We calculated decoded fNIRS reconstruction error metrics, as shown in Fig. 10, for each EEG frequency range and calculated patient wise reconstruction error. The gamma and beta frequency bands demonstrated the lowest error rates and in the lower EEG frequency ranges, we noticed increased fNIRS reconstruction error, possibly owing to the fact that our model was possibly not able to learn appropriate features to reconstruct fNIRS signals.

**Fig. 11:**
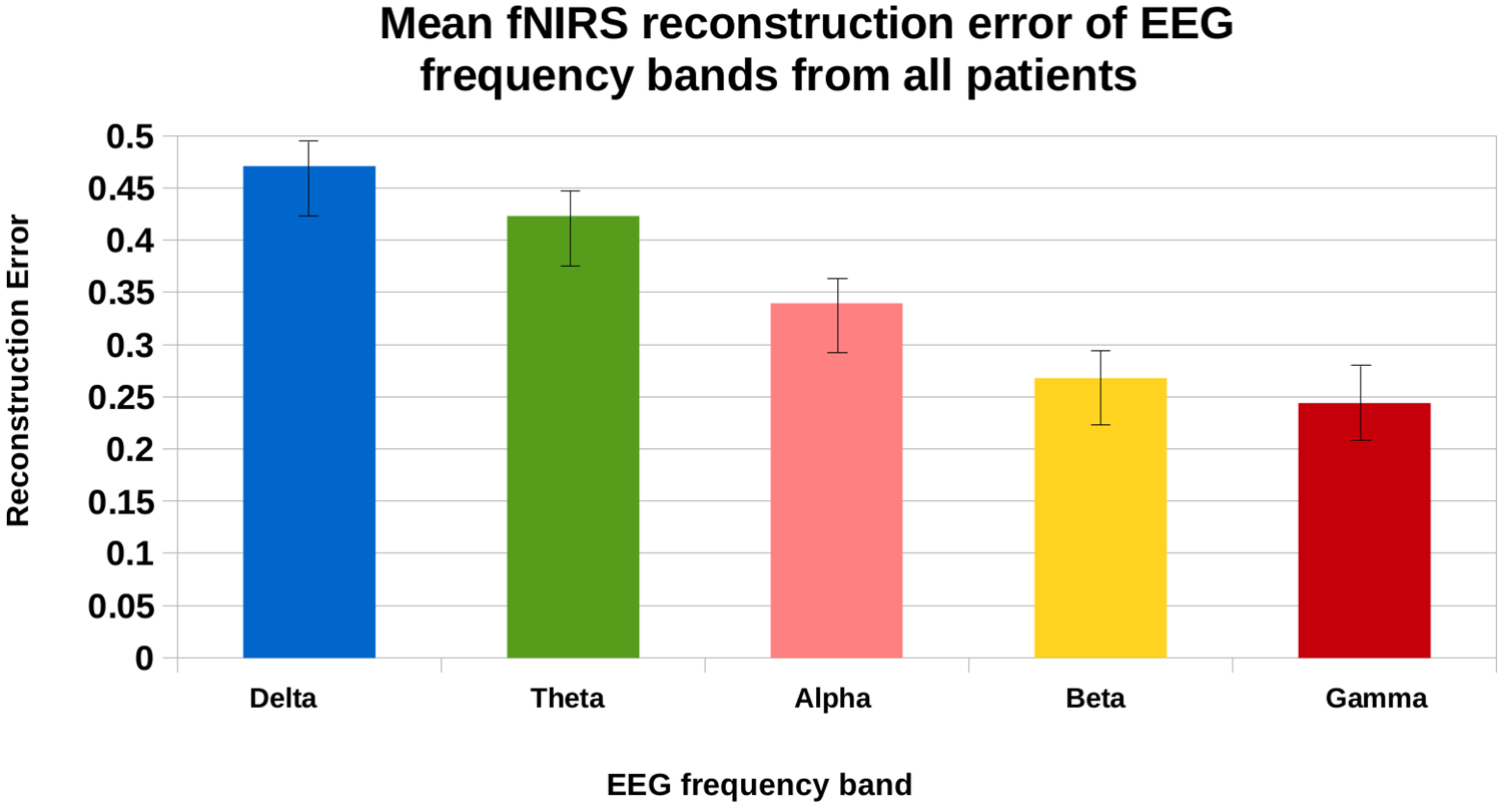
Mean fNIRS reconstruction error given specific EEG frequency ranges for all patients. The gamma range, which contains the greatest number of EEG frequencies, reconstructs with more fidelity and lowest reconstruction error metrics compared to other ranges with less frequency components

To further determine which EEG frequency band can reconstruct fNIRS signals with the lowest reconstruction error on average, we calculated band wise reconstruction error for all patients, as shown in Fig. 11. Following which, we conducted one-tailed paired t-tests to test whether there is a statistical difference in reconstruction error between any two of the five bands when compared to gamma in the following combinations: [delta, gamma], [theta, gamma], [alpha, gamma], and [beta, gamma]. Bonferroni correction was then applied to control the family-wise error rate to be less than 0.05. The gamma frequency band reconstructs fNIRS signals with increased fidelity on average as compared to other frequency bands.

### Functional Connectivity Results

**Fig. 12:**
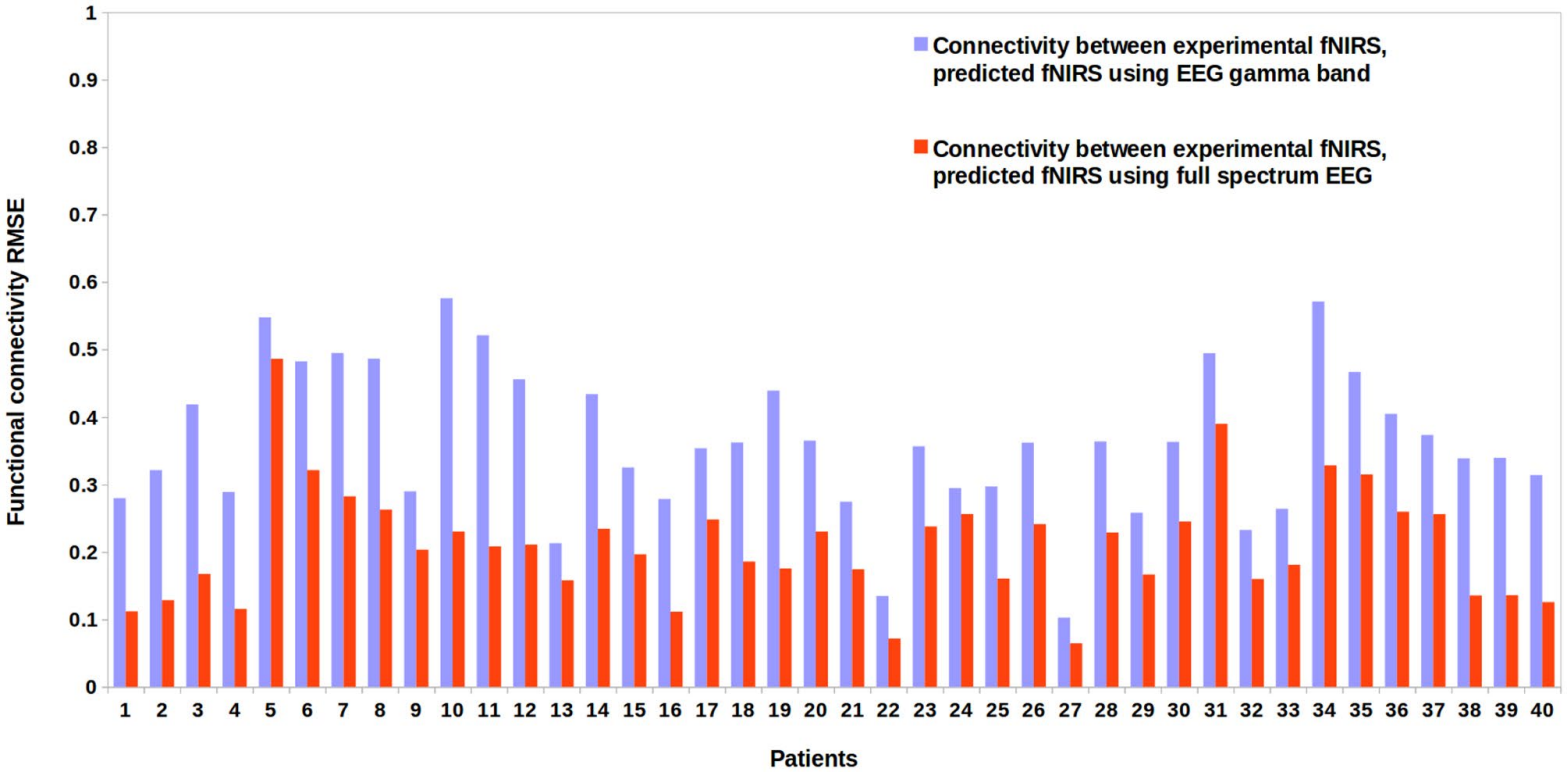
Estimator error (RMSE) for functional connectivity results for all patients. Error for connectivity analyses between experimental fNIRS and predictions using full spectrum and gamma band EEG signal input. The connectivity derived from the full spectrum EEG time series consistently has lower error compared to the connectivity derived from the gamma band

We computed functional connectivity mappings for experimental fNIRS and our model’s fNIRS reconstructions. We compared experimental fNIRS and fNIRS reconstructions derived from both full spectrum EEG and the EEG gamma band for all patients. The root mean square error (standard deviation of the residuals) was used as an estimator of the error in our connectivity studies. On a group level, we noticed a lower error in functional connectivity analyses between experimental fNIRS and fNIRS reconstructions derived from full spectrum EEG as compared with experimental fNIRS and fNIRS reconstructions derived from the gamma band and are shown in Fig. 12.

**Fig. 13:**
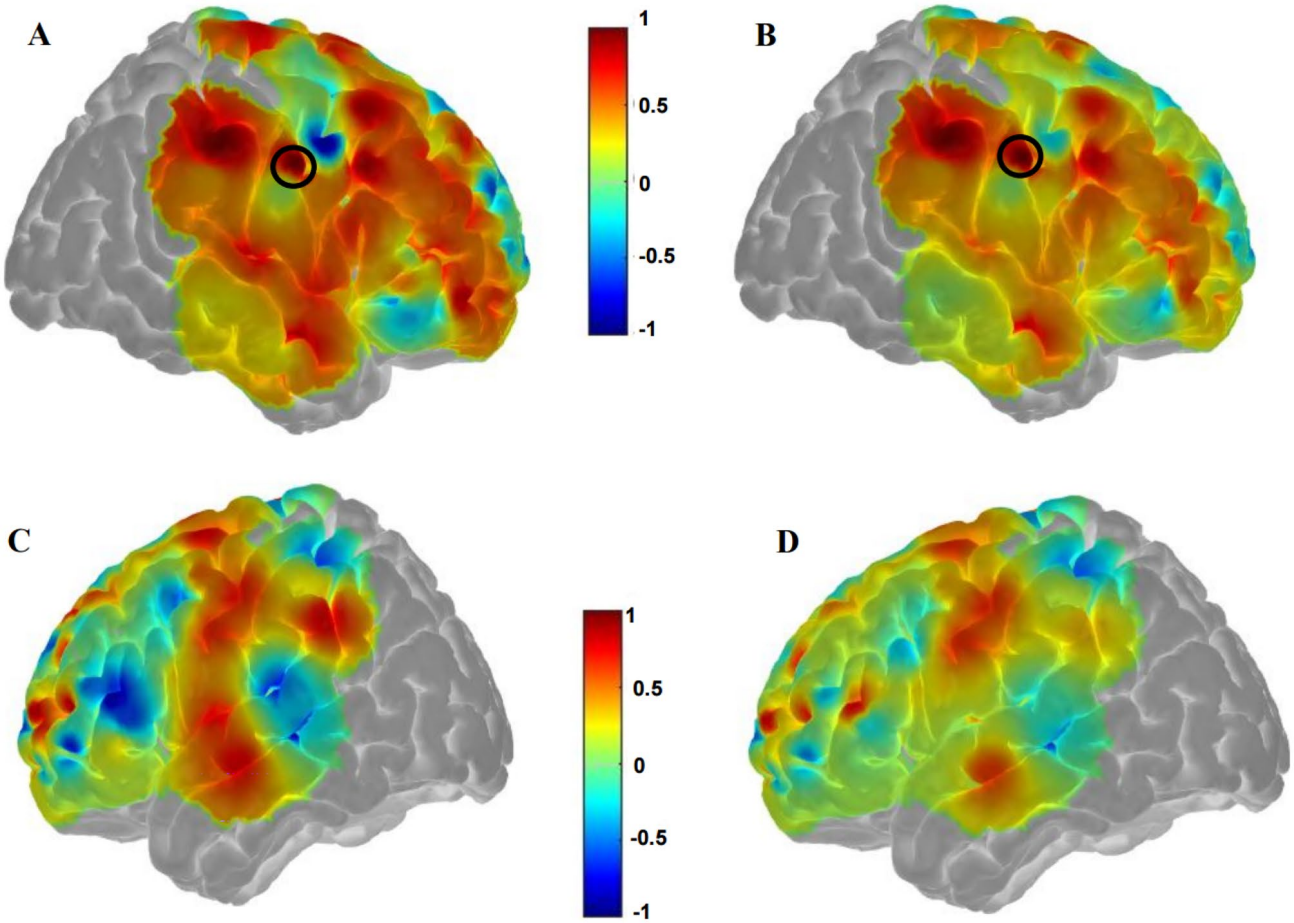
Functional connectivity results between experimental fNIRS and predicted resting state fNIRS using full frequency spectrum EEG as input for patient 22. We employed seed based functional connectivity analysis to obtain a surface brain map that describes brain functional connectivity correlation patterns. The seed region of interest (dark circle) is shown and full spectrum EEG was used as input into the model. Bilateral brain correlations using experimental **fNIRS (A, C)** and predicted resting state **fNIRS (B, D)** are shown. A and B display the right side of the brain, C and D display the left side of the brain. The connectivity profiles are seen to be similar between the maps generated using the experimental fNIRS results and the predictions of the model. A RMSE value of 0.07 corresponds to fNIRS signal reconstruction from experimental fNIRS and predicted fNIRS from full frequency spectrum EEG as model input

**Fig. 14:**
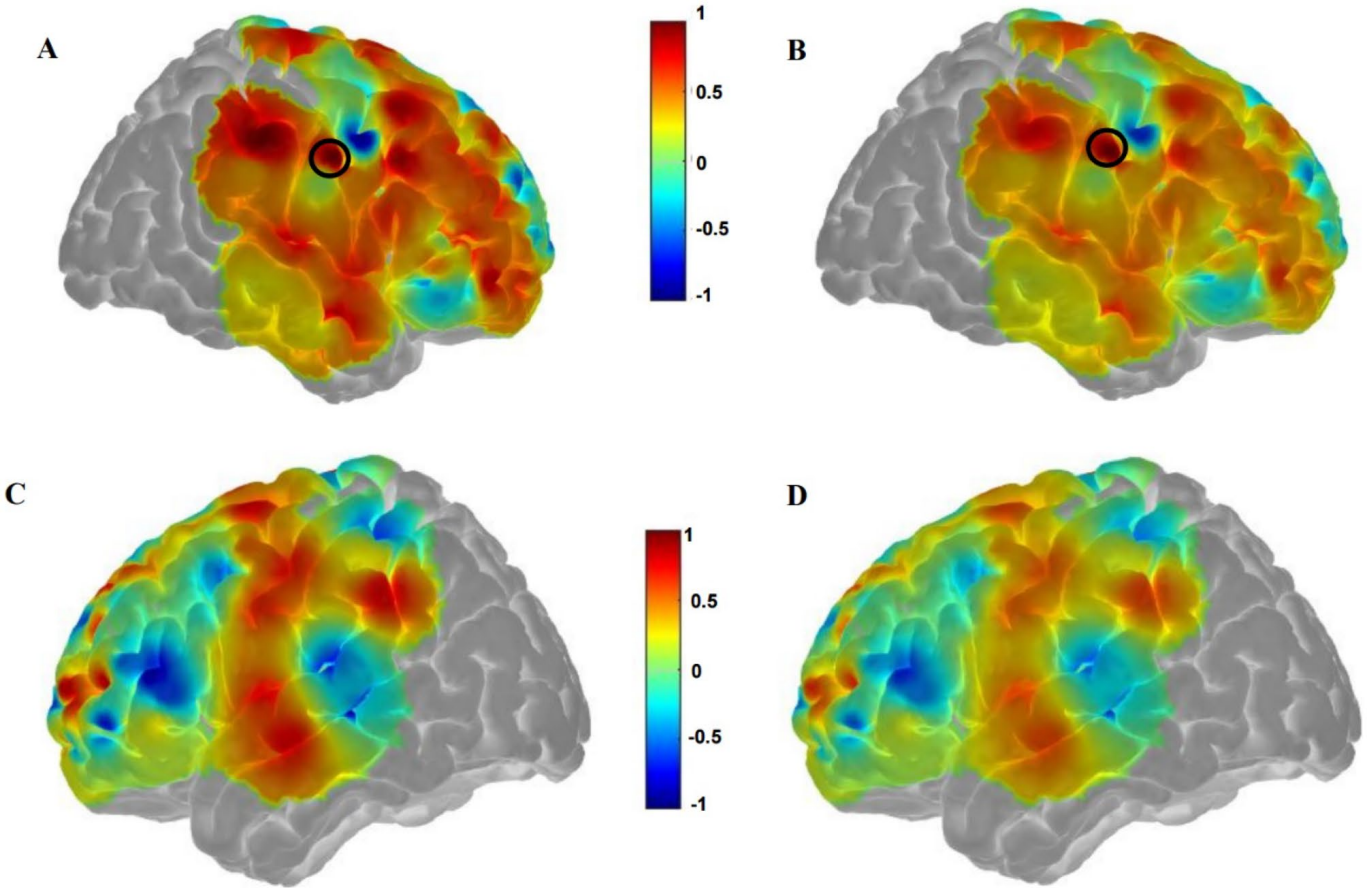
Functional connectivity results between experimental fNIRS and predicted fNIRS resting state using EEG gamma band as input for patient 22. Correlations from experimental fNIRS and predicted resting state fNIRS using EEG gamma band as input into our model are displayed. Bilateral brain correlations using experimental **fNIRS (A, C)** and predicted **fNIRS (B, D)** are shown. A RMSE value of 0.15 corresponds to fNIRS signal reconstruction from experimental fNIRS and predicted fNIRS from signals derived from the EEG gamma band as model input

Figures 13, 14 show examples of the functional connectivity mapping results generated using the correlations of the timeseries from patient 22, with the seed channel chosen as 20. First, we used full spectrum EEG predictions as input for functional connectivity computations (Fig. 13), followed by analysis using the EEG gamma frequency band as input (Fig. 14).

## Discussion

Deep learning models obviate cumbersome and brittle feature engineering processes replacing them with hierarchical feature learning. In this work, we developed a deep learning CNN-LSTM sequence-to-sequence autoencoder to predict fNIRS signals from resting state EEG signals in the epileptic brain. Our model was trained using a 60/20/20 split for training, testing, and validation, respectively. The results here demonstrate that in the context of epileptic resting state recordings, fNIRS signals can be predicted using full spectrum as well as specific frequency range EEG signals to a certain extent. We further validated our method by reconstructing the functional connectivity in the brain using the predicted fNIRS and compared it to the functional connectivity using experimental fNIRS.

From a neurophysiological standpoint, the resting epileptic brain is in a dynamic state and cerebral blood flow is in constant flux (Wang et al., 2011). Recent work has shown the presence of abnormal functional networks in the interictal state (Murta et al., 2015; Richardson, 2012). Thus, even with removal of systemic physiological components underlying compensation by molecular and cellular mechanisms can possibly help predict components of systemic physiology in addition to hemodynamic brain activity (Pressl et al., 2019). Our experimental findings can be related to known physiological phenomena being generated at the frequency of Mayer waves (~0.1 Hz), as these oscillations reflect fluctuation in cerebral arterial blood pressure (Nikulin et al., 2014; Schwab et al., 2009). The presence of these oscillations persisting after filtering can be partly due to the fact that they share a common spectral range with typical hemodynamic responses (Yücel et al., 2016). On the other hand, these oscillations correspond to cerebral vasomotion (i.e., extra neuronal) and are possibly related to blood vessel tonal oscillation (Aalkjær et al., 2011; Julien, 2006; Quaresima & Ferrari, 2019; Sassaroli et al., 2012).

The exact mechanics of physiological signal presence within EEG signals has not been established with certainty. However, experimental results from this work suggest the following: our model can capture subtle hemodynamic dependencies within the EEG resting state signal and its fNIRS correlate via the neurovascular coupling phenomenon.

These nuanced features within the EEG signal are encoded and subsequently decoded by the architectural components of the model, particularly the convolutional LSTM parameters (Greff et al., 2017; Sutskever et al., 2014). The model’s encoder and decoder and parameters (e.g., the activation function) may have enhanced feature extraction in resting state EEG data and its corresponding correlate in fNIRS signals. In addition, the features computed by using the outputs or hidden states of the recurrent units and the model may extract long-term dependencies (electrical and/or physiological) in resting state EEG signals from the LSTM modules via the gating mechanism (Sutskever et al., 2014). Furthermore, when cerebral blood flow (CBF) varies, changes occur in both the metabolic and electrical activity of cortical neurons with corresponding EEG changes (Sassaroli et al., 2012).

Events responsible for evoking the fNIRS response can be divided into subthreshold synaptic and suprathreshold spiking activities (Curtin et al., 2019; Sharbrough et al., 1973). Excitatory and inhibitory neurons which are often located within close proximity in the brain are simultaneously active and may contribute to the hemodynamic response (Franaszczuk et al., 2003). Slower EEG frequency envelopes (i.e., delta and theta) are generated by the thalamus and cortical cells in layers II-VI. Faster frequencies (i.e., beta and gamma) arise from cells in layers IV and V of the cortex (Foreman & Claassen, 2012; Merker, 2016). Changes in electrical potential seen in EEG recordings are closely tied to cerebral blood flow (CBF) and when normal CBF declines to approximately 25– 35 ml/100 g/min, the EEG signal first loses faster frequencies, then as the CBF decreases to approximately 17–18 ml/100 g/min, slower frequencies gradually increase. The interdependent relationship between CBF and neuronal activity in the resting epileptic brain is theorized to be captured by the model used in this work. Exploring the spatial localization of EEG frequency oscillations can help to determine if the presence of physiological signals is variable across patients and electrodes thereby possibly lending credence to the hypothesis that these oscillations are unlikely to be generated by a single source.

We show spatial decoding is possible using our model. Examination of the LSTM memory units and the latent space architecture in autoencoders can demonstrate correlation between data that were previously unknown. Utilizing the architecture developed here to predict brain hemodynamics, a next step would be to understand the structure of the latent variable (multidimensional vector) to unpack the principal components of the fNIRS or EEG signal.

A second point for further investigation is to integrate an attention mechanism in our model. Since LSTM cells can lead to ambiguous memory activations, an attention mechanism allows for encoding input into a sequence of vectors and from this, we can choose a subset adaptively during decoding. In this condition, the model no longer needs to utilize fixed length vectors thereby increasing performance metrics at the cost of computational time. Attention implemented in our model would enable us to inspect the relationship between encoded and decoded sequences by model weight visualization.

In comparison with lower frequency range EEG signals, results here suggest that higher frequency EEG envelopes reconstruct fNIRS signals with less error. Our results corroborate that EEG gamma band based fNIRS reconstructions show a closer fit between the observed and predicted hemodynamic responses as opposed to other EEG frequency ranges (Ebisch et al., 2005; Murta et al., 2015; Niessing et al., 2005). This is possibly because higher frequencies engage an increased number of neurons, but it is less apparent if this is attributed to baseline network activity or part of a pivotal functional role. Gamma rhythms in the brain provide an indication of engaged networks and have been observed in several cortical and subcortical structures. These rhythms are typically stronger for some stimuli as compared to others, thereby displaying selectivity to that of nearby neuronal activity (Jia & Kohn, 2011; Whittingstall & Logothetis, 2009). GABA-ergic inhibitory interneuron activity is considered to be crucial to generate EEG gamma frequency activity and this may be increased via interactions with excitatory neurons (Jia & Kohn, 2011; Park et al., 2011; Ray & Maunsell, 2010). However, to fully interpret the impact of this activity warrants an investigation into the cellular mechanisms responsible for their generation.

In the second part of our work, we explored functional connectivity in the resting state of the epileptic brain. We hypothesized that our network’s predictions can help reveal functional connections and on a group level, predicted fNIRS from full spectrum EEG have higher connectivity as compared to predictions derived from the EEG gamma band. Experimental resting state fNIRS data and predicted fNIRS data was correlated to reveal similar connections near the set seed but metrics decreased generally as distance increased from the seed. This can be due to numerous factors: 1. noise causing a decrease in reconstruction quality, 2. a decrease in gamma activations at the region of interest, and 3. model parameters unable to completely learn the nuances present within the signal. Furthermore, systemic artifacts from the scalp and skull behave as dominant noise sources in resting state fNIRS signals, leading to inaccurate reconstruction. Utilizing an EEG-fNIRS experimental setup with short separation channels, measuring approximately 1–2 cm in spatial separation between source and detector could lead to sufficient noise reduction and improved signal sensitivity (Gagnon et al., 2012; Kohno et al., 2007). We hypothesize that reconstruction metrics and corresponding functional connectivity network measures stabilize with increased signal quality and resting state duration, thereby decreasing the disparities present between experimental and predicted time series.

The resting state epileptic brain and connectivity between brain networks is dynamic (Deco et al., 2011; McKenna et al., 1994). Typically, fMRI has been used for computing functional connectivity but there are inherent limitations of fMRI, particularly, slow dynamics, regional variability of the hemodynamic response to neuron firing and the fact that some patients are not able to undergo an fMRI scan easily (i.e., claustrophobia, paroxysmal seizure occurrence during scanning) (Pressl et al., 2019; Richardson, 2012). By showing the possibility of obtaining brain hemodynamic data from neural signals, the results here add an additional dimension for understanding the epileptic human brain, aid in clinical decision making, and provide a complementary measure to fMRI, particularly in locations where access to fMRI technology is scarce or not possible.

Scalp EEG technology remains the clinical gold standard for the noninvasive assessment of electrical brain activity (Dash et al., 2017). Using EEG signals in conjunction with predicted brain hemodynamics can possibly improve clinical management and ultimately patient outcomes (Connolly et al., 2015; Helbok & Claassen, 2013). Multimodal EEG-fNIRS analysis using deep learning frameworks, as the one presented in this work, can improve our understanding of cerebral neurovascular coupling and pathophysiology. The results from this work can be abstracted for applications to other neurological and neuropsychiatric pathologies, such as stroke, spinal cord injuries, traumatic brain injuries, Alzheimer’s disease, attention-deficit hyperactivity disorder, post-traumatic stress disorder, and dementia to name a few (Fair et al., 2013; Phillips et al., 2018; Siegel et al., 2016). Furthermore, hemodynamic predictions from electrical brain signals can be useful in treatment strategies utilizing neurofeedback (i.e., neuroprosthetics, transcranial direct current stimulation) as well as towards developing precision medicine strategies (DeBettencourt et al., 2015; Dutta et al., 2015; Kotliar et al., 2017; Nicholson et al., 2016; Ros et al., 2014; Sitaram et al., 2017; Thair et al., 2017). Predicting hemodynamics from EEG increases clinical diagnostic specificity, allowing differentiation between pathological conditions that may appear similar but require different treatments (Citerio et al., 2015; Le Roux, 2013). Currently, therapeutic strategies follow a ‘reactive’ model: corrective actions are triggered by abnormal values in single parameters (i.e., EEG signals) and a stepwise approach is used with increasing therapeutic intensity. Comprehensive signals (i.e., EEG and predicted hemodynamics) can shift this paradigm towards a ‘goal-directed’ management strategy (Le Roux, 2013; Maas et al., 2012; Schmidt & De Georgia, 2014).

## Conclusion

We designed and implemented a deep learning model to predict resting state hemodynamics given specific resting state scalp EEG frequencies from a cohort of epileptic patients. The robust multidimensional dataset used here allowed us to investigate the relationship between brain hemodynamics and neural signals via neurovascular coupling. Using a deep learning architecture, we performed a thorough analysis of each EEG frequency range and its complementary fNIRS prediction; further we analyzed functional connectivity between brain regions using frequency range predictions. We noted that higher EEG frequency bands provided hemodynamic predictions with the highest metrics.

## Information Sharing Statement

Sharing the data used in this study is bound by the ethics of the institutional review boards of Sainte-Justine Hospital and Centre Hospitalier de l’Université de Montréal which approved the study. The custom code used in this study is available upon reasonable request.
